# Empyema Resulting From a Nephro-Pleural Fistula in an Adult With Polycystic Kidney Disease With Obstructive Uropathy

**DOI:** 10.7759/cureus.57671

**Published:** 2024-04-05

**Authors:** Kundan Mehta, Sidhaant Nangia, Rhea Gandhi, Spandana Chaudhury

**Affiliations:** 1 Respiratory Medicine, Dr. D. Y. Patil Medical College, Hospital & Research Centre, Pune, IND

**Keywords:** renal calculi, adpkd patients, pleural empyema, obstructive uropathy, perinephric abscess

## Abstract

Nephropleural fistula, a rare complication of percutaneous nephrolithotomy (PCNL), occurred in a 45-year-old male with adult autosomal dominant polycystic kidney disease (ADPKD). The patient had undergone right PCNL in 2021 and 2023 and presented to the emergency department with symptoms of fever, breathlessness, and cough lasting one week. Imaging studies, including chest radiograph and contrast-enhanced computed tomography (CECT) of the abdomen and pelvis, revealed gross right pleural effusion, right perinephric abscess, multiple renal cysts, right renal calculi and right ureteric calculi causing severe right hydronephrosis and proximal hydroureter. The imaging also confirmed a nephropleural fistula, with the right kidney's perinephric abscess communicating with the right pleura via the right subhepatic space. Subsequent thoracic ultrasound showed a large effusion of 1500ml with underlying lung collapse. Diagnostic thoracocentesis confirmed empyema, necessitating immediate tube thoracostomy. CT intravenous urography confirmed a non-functioning right kidney. The perinephric abscess was drained with a PCNL tube and meanwhile, pleural fluid and perinephric abscess isolated Klebsiella pneumonia on cultures. The patient received parenteral antibiotics and intravenous fluids and had an intercostal drain and PCNL tube in place for drainage. A right nephrectomy was recommended due to the non-functioning right kidney and the patient is awaiting the procedure.

## Introduction

Nephropleural fistula, although uncommon, may arise as a complication following the percutaneous nephrolithotomy (PCNL) procedure. The pelvicalyceal system and pleural cavity communicate directly and persistently in this state [1]. Trauma resulting from percutaneous renal access is often implicated as a probable cause for the development of such a fistulous connection [2]. Typical symptoms include shortness of breath, pleuritic chest pain, and pleural effusion. Diagnosis of nephropleural fistula typically involves the use of contrast studies to confirm the tract, alongside biochemical analysis. Treatment primarily revolves around managing both the pelvicalyceal system and any associated pleural fluid collection.

## Case presentation

**Figure 1:**
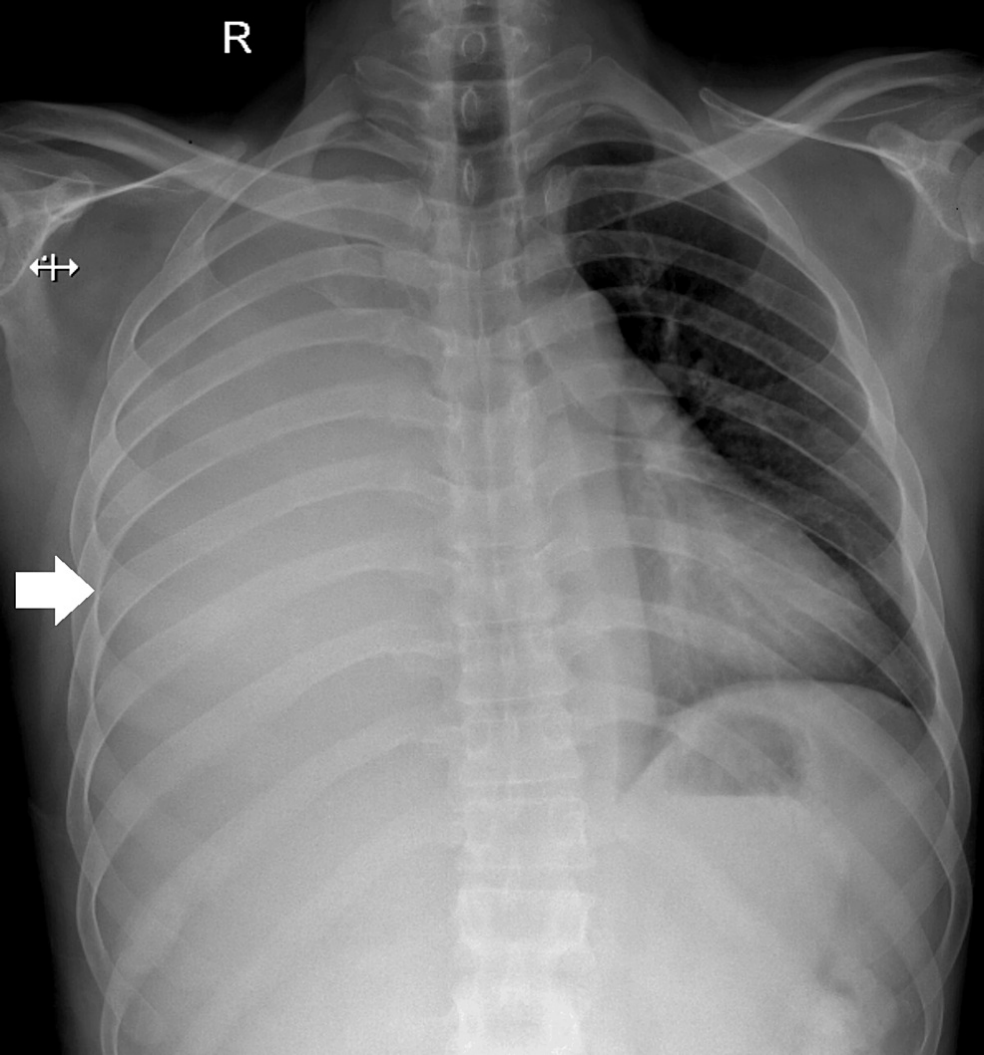
Chest radiograph with significant right pleural effusion (white arrow) with mild mediastinum shift to the left

**Figure 2:**
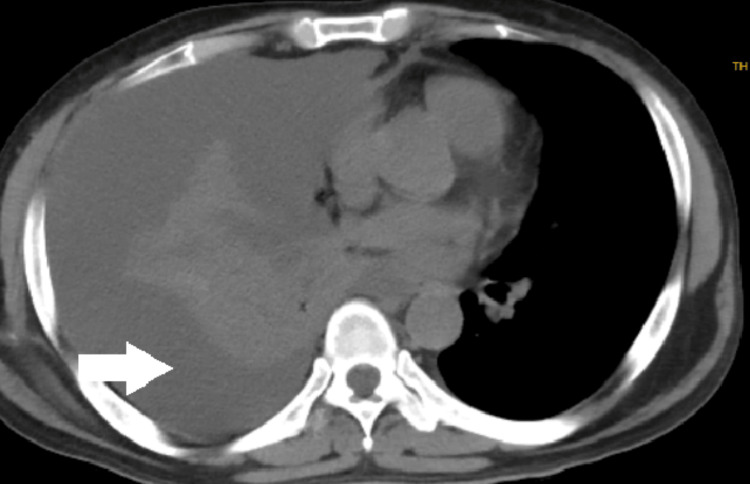
Chest CT-gross right pleural effusion (white arrow) with mild mediastinum shift to left with underlying lung collapse

A 45-year-old male with adult autosomal dominant polycystic kidney disease (ADPKD), with a history of multiple renal calculi and a previous right PCNL in 2021 and 2023, presented to the emergency department complaining of fever, breathlessness, and cough persisting for one week. An immediate chest radiograph and CT chest revealed significant right-sided pleural effusion (Figures 1, 2).

**Figure 3:**
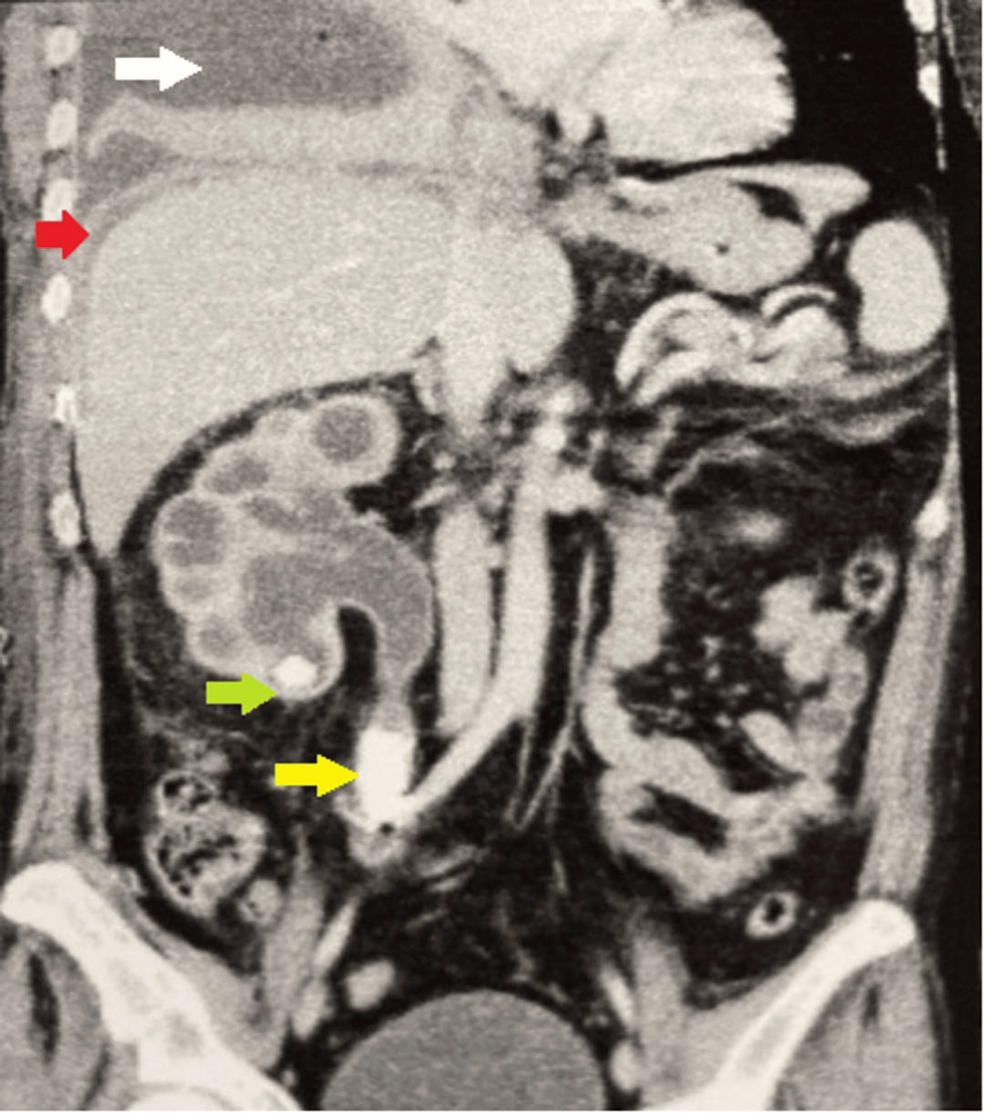
CECT abdomen pelvis (coronal view) renal (green) and ureteric calculus (yellow) with minimal ascites (red arrow) and right empyema (white arrow)

**Figure 4:**
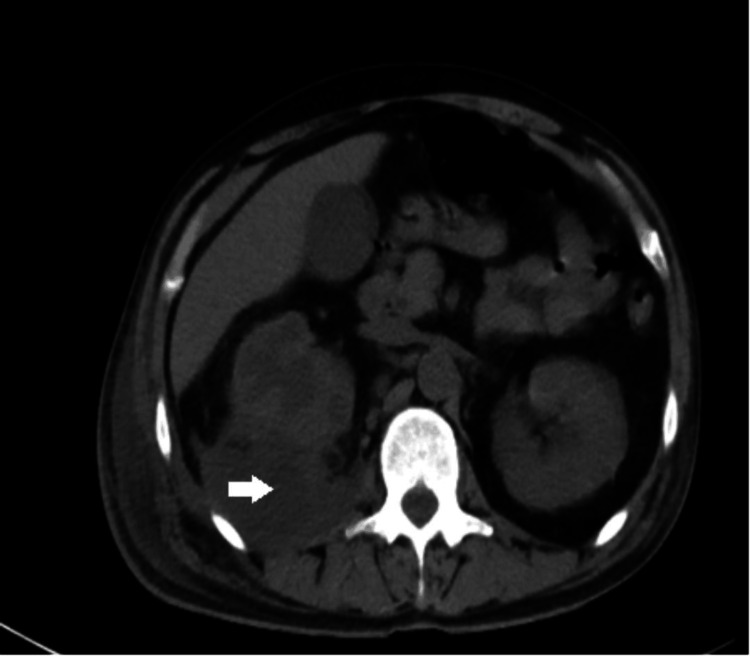
CT abdomen (axial view) - right perinephric abscess (white arrow)

Given his abdominal symptoms and history of PCNL, a contrast-enhanced computed tomography scan of the abdomen and pelvis was performed, revealing a right perinephric abscess, multiple right renal cysts, right renal calculi, and right ureteric calculi causing marked hydronephrosis and proximal hydroureter (Figures 3, 4).

**Figure 5:**
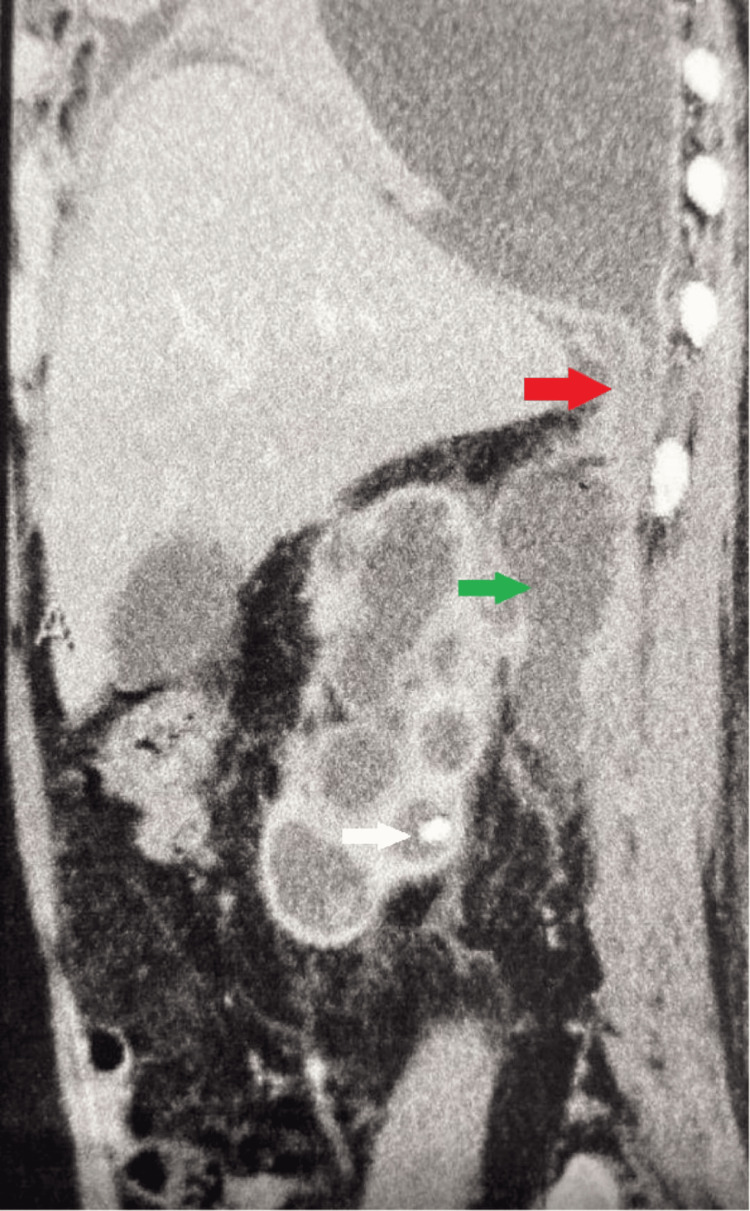
CECT abdomen (sagittal view) - renal calculus (white arrow), perinephric abscess (green arrow), and nephropleural fistula (red arrow) CECT: Contrast-enhanced computed tomography

Furthermore, a nephropleural fistula, with the right kidney's perinephric abscess communicating with the right pleura via the right subhepatic space was noted (Figure 5).

**Figure 6:**
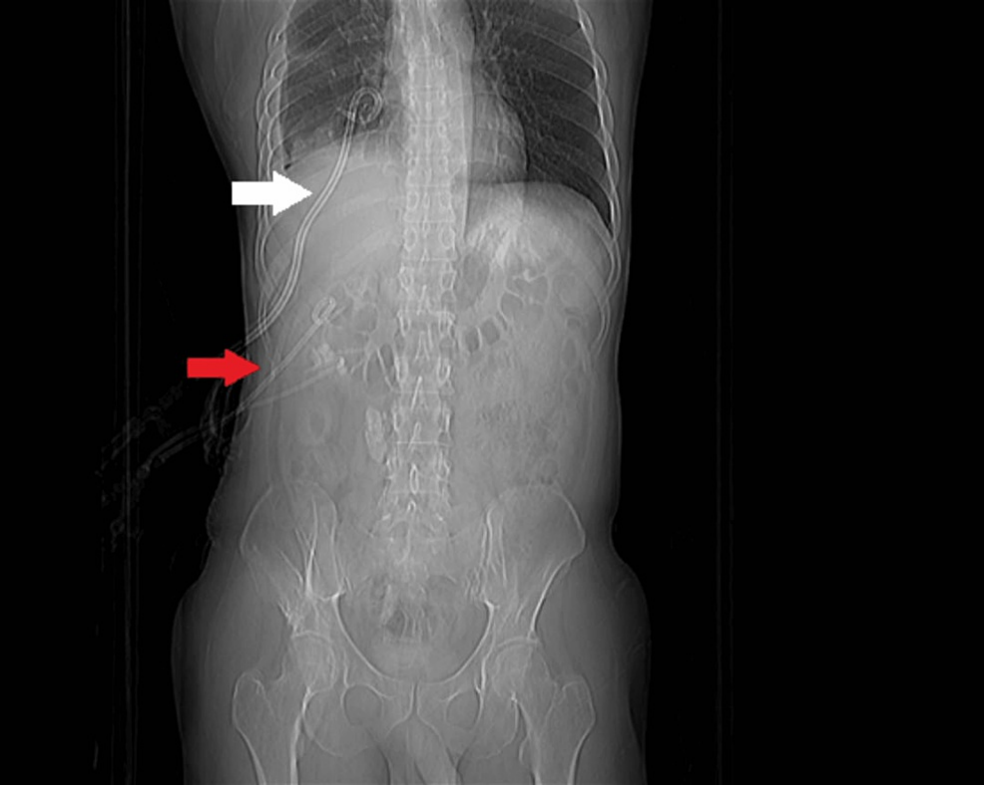
CT topogram chest and abdomen showing right chest tube (white arrow) and right renal drain (red arrow)

Subsequently, the patient was admitted, and diagnostic thoracocentesis yielded yellowish, turbid fluid with an exudative, neutrophilic-predominant biochemical analysis, indicative of empyema by Light's criteria. An immediate right tube thoracostomy was performed. The perinephric abscess was dealt with a PCNL drain (Figure 6).

**Figure 7:**
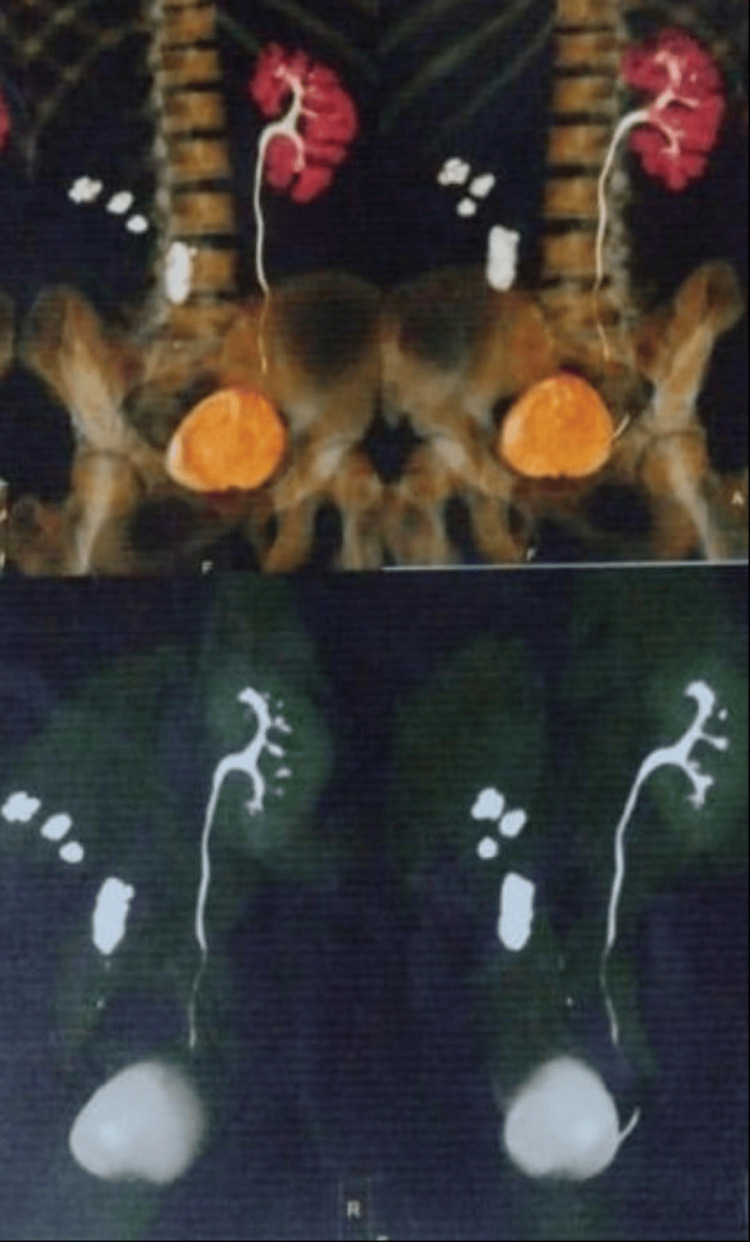
CT pyelography showing non-functioning right kidney with renal and ureteric calculi

Cultures from both the pleural effusion and perinephric abscess revealed *Klebsiella pneumoniae*, a Gram-negative bacterium. The patient received parenteral antibiotics and intravenous fluids. Approximately 1500cc of pus was drained via the right pleural space and around 120cc from the perinephric space. Both the intercostal drainage tube and PCNL drain were removed after one month. Follow-up ultrasound of the thorax and abdomen showed minimal, non-significant fluid in the right pleural space and no perinephric collection and the patient was advised to undergo a right nephrectomy in view of the non-functioning right kidney as confirmed on CT intravenous urography (IVU) (Figure 7).

## Discussion

Empyema thoracis is an infectious condition that causes pus to build up in the pleural space. It usually results from the spread of infection from a proximal source and is frequently linked to lung infections. Even though extra-pulmonary empyema is uncommon, cases resulting from a fistula connecting the thorax and urinary system are extremely unusual clinical phenomena, even though up to 20% of renal infections may have pulmonary consequences [2]. Empyema is characterized by the macroscopic presence of leukocytes and the appearance of thick, turbid pleural fluid. The difference between effusion and empyema is based on the concentration of leukocytes. The following factors are used by Light’s criteria to diagnose empyema: low glucose, high lactate dehydrogenase (>1000U/L), polymorphonuclear predominance in the exudate/pus, positive Gram stain for organisms, pH < 7.2, and low glucose levels [3]. Conditions that look radiopaque and non-specific in the lower lung fields on chest radiographs, such as atelectasis, pleural effusions, pneumonia, raised hemidiaphragm, and lung or pleural masses with direct pleural surface contact, can be quickly distinguished using ultrasound [4]. CT imaging with contrast enhances the ability to see the distinctive "split pleural sign," which is defined as thicker, more pronounced parietal and visceral pleural layers divided by pleural fluid. In contrast to parapneumonic effusions, pleural thickening and enhancement are more frequently seen in empyemas and are frequently linked to increased pleural thickness [5]. The buildup of pus within an obstructed collecting system, usually under pressure, is the hallmark of pyonephrosis. Stricture, stone, congenital anomaly, tumor, or other factors may cause the obstruction. Catheterization, medical device placement, endoscopic procedures, renal failure, kidney transplantation, or acquired/congenital immune system deficiencies are some of the other causes that may be linked to the obstruction [6]. CT imaging is useful in detecting renal and perinephric abscesses, which frequently spread into the iliopsoas muscle and retroperitoneal regions. It can also assess whether fistulae that link to the colon, duodenum, or pleura are present. Usually, Gerota's fascia and the renal capsule are affected by a perinephric abscess. The rarity of pleural empyema in ADPKD related to a nephropleural fistula is underscored by the lack of known cases of pleural empyema resulting from ascending urological infections that cause pleural involvement. A nephropleural fistula is an uncommon and frequently disregarded complication of PCNL. It is defined by a direct and continuous connection between the intrathoracic cavity and the pelvicalyceal system. Using a perinephric catheter to decompress the collecting system and drain the pleural cavity is necessary for managing such fistulae. Additionally, it's crucial to identify and address any obstruction in the nephro-ureteric system if present [7]. In our patient's case, treatment included drainage of the perinephric abscess and empyema, along with the administration of appropriate parenteral antibiotics to control infection. Furthermore, plans were made for a nephrectomy due to the confirmed non-functional right kidney as revealed on CT IVU. Nephropleural fistula incidence in PCNL was reported to be 0.87% in a comprehensive retrospective investigation. The literature has provided a thorough description of nine cases of nephropleural fistula following PCNL. It is likely underreported, though, and its frequency is predicted to increase as percutaneous urologic procedures become more common. A lower body mass index due to perirenal fat loss, intercostal approaches above the 11th rib, and younger age are major risk factors for nephropleural fistula [8].

## Conclusions

Iatrogenic nephropleural fistula is an infrequent yet perilous complication of PCNL. Doctors should be particularly vigilant for nephropleural fistula in patients who develop pleural effusion following PCNL, especially if gram-negative bacteria are identified in the pleural fluid culture and for them to determine the correct course of diagnosis for immediate disease-specific treatment to be facilitated. Timely recognition, comprehensive diagnostic evaluations, and targeted therapeutic approaches are instrumental in facilitating swift recuperation for the patient.
